# Effect of Testosterone on Neuronal Morphology and Neuritic Growth of Fetal Lamb Hypothalamus-Preoptic Area and Cerebral Cortex in Primary Culture

**DOI:** 10.1371/journal.pone.0129521

**Published:** 2015-06-08

**Authors:** Radhika C. Reddy, Rebecka Amodei, Charles T. Estill, Fred Stormshak, Mary Meaker, Charles E. Roselli

**Affiliations:** 1 Department of Physiology and Pharmacology, Oregon Health and Science University, Portland, Oregon, United States of America; 2 Department of Animal and Rangeland Sciences, Oregon State University, Corvallis, Oregon, United States of America; 3 Department of Clinical Sciences, College of Veterinary Medicine, Oregon State University, Corvallis, Oregon, United States of America; Institut National de la Recherche Agronomique-CNRS UMR6175, FRANCE

## Abstract

Testosterone plays an essential role in sexual differentiation of the male sheep brain. The ovine sexually dimorphic nucleus (oSDN), is 2 to 3 times larger in males than in females, and this sex difference is under the control of testosterone. The effect of testosterone on oSDN volume may result from enhanced expansion of soma areas and/or dendritic fields. To test this hypothesis, cells derived from the hypothalamus-preoptic area (HPOA) and cerebral cortex (CTX) of lamb fetuses were grown in primary culture to examine the direct morphological effects of testosterone on these cellular components. We found that within two days of plating, neurons derived from both the HPOA and CTX extend neuritic processes and express androgen receptors and aromatase immunoreactivity. Both treated and control neurites continue to grow and branch with increasing time in culture. Treatment with testosterone (10 nM) for 3 days significantly (P < 0.05) increased both total neurite outgrowth (35%) and soma size (8%) in the HPOA and outgrowth (21%) and number of branch points (33%) in the CTX. These findings indicate that testosterone-induced somal enlargement and neurite outgrowth in fetal lamb neurons may contribute to the development of a fully masculine sheep brain.

## Introduction

Exposure of the vertebrate brain to testosterone during a critical period of fetal development establishes permanent sex differences in reproductive physiology as well as a broad spectrum of behaviors and cognitive functions [1–3]. Sex steroids are also responsible for sexual dimorphisms in brain regions that regulate these functions [4,5]. For example, sexual dimorphisms in the volume of the medial preoptic area (MPOA) occur in several species, in particular the central region of the medial preoptic nucleus, which has been named the sexually dimorphic nucleus of the preoptic area (SDN-POA) in rats where it was first described [6]. The SDN-POA of males is larger in volume than that of females. This correlates with quantitatively greater measures of copulatory behavior exhibited by males [7,8]. This difference in nuclear volume has been attributed to the presence of more neurons in the SDN-POA of males than of females [9]. Gonadal steroids determine neuron number in part by controlling the occurrence of cell death during brain sexual differentiation in rodents [10–12]. In addition, gonadal steroids have direct effects on soma size and the dendritic growth, which also contribute to volume differences in the SDN-POA [9,13–15].

In the sheep, a cluster of neurons exists bilaterally in the central portion of the medial preoptic area. This structure is called the ovine sexually dimorphic nucleus (oSDN) because it is approximately two times larger and contains more neurons in rams that sexually prefer ewes than in ewes or in rams that prefer other rams as sexual partners[16]. The oSDN develops prior to birth and is enlarged in genetic females by exposure to exogenous testosterone during a prenatal critical period that occurs after the external genitalia have differentiated [17,18]. Exposure of adult sheep to exogenous testosterone does not affect oSDN volume, confirming that nuclear size is programmed prenatally [19]. The cellular mechanism whereby testosterone controls the development of the oSDN is not yet established. In contrast to rodents, gonadal steroids do not appear to modulate cell death (apoptosis) within the developing oSDN [20]. Testosterone has been shown to increase the volume of neuropil in motor neurons [21,22], and may function in a similar manner in oSDN.

The present study was conducted to test the hypothesis that testosterone effects neuronal soma and dendrite morphology, which could in turn contribute to the development of a fully masculine sheep brain. Thus, cells derived from the hypothalamus-preoptic area (HPOA) and cerebral cortex (CTX) of lamb fetuses were grown in primary culture and studied to examine the direct effects of testosterone on soma size, total neuronal process length, the number of neuronal processes and the number of process branch points.

## Materials and Methods

### Ethics Statement

Animal procedures complied with the National Institutes of Medicine *Guide for the Care and Use of Laboratory Animals* and were approved by the Institutional Animal Care and Use Committee of Oregon State University (protocol number: 4331). All surgeries were carried out under general anesthesia induced with ketamine hydrochloride (4.4 mg/kg) plus diazepam (0.1 mg/kg) and maintained during surgery with oxygen:isoflurane mixture.

### Animals

Lamb fetuses were obtained from pregnant Polypay ewes that were bred and maintained under standard husbandry conditions at the AAALAC-approved Sheep Research Facility at Oregon State University in Corvallis, OR. Eight fetuses (6 males and 2 females) were delivered surgically from 5 dams at gestational day (GD) 53 as described previously [17]. Blood samples were collected from the umbilical artery and fetuses were sexed by examination of their external and internal genitalia.

### Primary Cultures and Cell Treatments

Cells obtained from the brains of GD 53 lamb fetuses were cultured separately according to sex. Cells of brains at this stage of gestation were chosen because previous studies demonstrated that high yields of neurons are obtained from fetal sheep cerebral cortex between 7 and 9 weeks of age [23–25]. This constitutes the main phase of neuronal proliferation preceding the phase of glial cell proliferation when the fetal lamb brain is similar in maturity to those of neonatal rodents [26,27]. Brains were removed from the calvarias into ice-cold Hibernate E media (BrainBits; Springfield, IL). The meninges were removed and the hypothalamus-preoptic area (HPOA) and cerebral cortex (CTX) dissected. The HPOA was removed with iridectomy scissors as a 2 mm deep block of tissue that was limited rostrally by the optic chiasm, caudally by the mammillary bodies and laterally by the lateral hypothalamic sulcus. CTX cells were derived from bilateral dissections of the frontal and parietal lobes.

Cells were cultured using an adaptation of the BrainBits online protocol (http://www.brainbitsllc.com/dissociated-primary-neuronal-plating-protocol/). In brief, glass coverslips (Carolina Biological; Burlington, NC) were pre-incubated with Poly-L-Lysine (Sigma-Aldrich; St. Louis, MO), washed thoroughly with molecular grade water (HyClone), placed into 2.3 cm^2^ multiwell tissue culture plates (Corning Inc. Life Sciences, Tewksbury, MA) and incubated overnight at 37°C in phenol-free Neurobasal Media (Invitrogen, Life Technologies, Grand Island, NY) supplemented with serum free B-27 supplement (InVitrogen). Fetal lamb brain tissues were dissociated into single cells by digestion for 20 minutes at 37°C with 0.2% papain (Worthington Biochemical Corp.; Lakewood, NJ) and 0.1% DNase I (Invitrogen) in Ca^2+^Free Hibernate E buffer (BrainBits), sequentially washed in Hibernate E supplemented with B-27 followed by mechanical trituration with fire polished Pasteur pipettes. Cells were then plated on glass cover slips at a density of 300,000 cells per 2.3cm^2^ well for the cortical cultures and 400,000 cells per 2.3cm^2^ well for the HPOA cultures, 2–3 wells per condition. After 1 hr., half of the medium was replaced with fresh phenol red-free Neurobasal medium supplemented with B-27 and GlutaMAX I (Gibco). For time-course analysis, cells were maintained *in vitro* for 2–5 days. For morphometric analysis, cells were maintained for 3 days *in vitro* (DIV) under 3 conditions: no treatment; vehicle treatment (ethanol; 1:2,000 final dilution) and testosterone treatment (10 nM final concentration; Steraloids; Newport, RI). This concentration of testosterone was chosen because it is physiological and has been shown previously to stimulate neurite outgrowth in primary hypothalamic neurons derived from rats [15]. As described above, media was changed 1 hr. after plating. For treatments, a stock solution of testosterone (20mM) was made in 100% ethanol. On the day of drug treatment, stock testosterone or ethanol vehicle were diluted (1/1000) in feeding media and used to replace 50% of the media resulting in treatment with testosterone (10nM final concentration) or ethanol vehicle (0.05% final concentration). Cultured neurons were incubated at 37°C in an atmosphere of 95% air– 5% CO_2_.

### Immunocytochemistry

Cells were fixed in freshly made 4% paraformaldehyde in 10 mM phosphate-buffered saline (PBS; pH 7.4) for 20 minutes at room temperature, permeabilized with 0.2% Triton-X in PBS for 10 min, blocked in 10% bovine serum albumin (BSA) in PBS (BSA/PBS) for 1 hr and then incubated overnight at 4°C with primary antibodies diluted in 3% BSA/PBS. Immunocytochemistry was performed using the following primary antibodies: a mouse monoclonal anti-neuronal class III β-tubulin antibody (diluted 1:1000, Covance TUJ1, Covance Inc.; Princeton, NJ), PG-21 rabbit polyclonal anti-androgen receptor antibodies (1:100, Upstate 06–680; EMD Millipore; Billerica, MA) and rabbit polyclonal anti-aromatase antibodies (1:20, Acris Antibodies Inc, SanDiego, CA). All 3 antisera have been shown to specifically recognize their target antigen in sheep tissue [25,28,29]. Immunoreactions were visualized with Jackson Immunological FitC anti-mouse (1:1,000 dilution) or Cy3 anti-rabbit (1:500 dilution) in 3%BSA/PBS at room temperature for 45 min. Cells were mounted in Prolong Gold antifade reagent with DAPI (InVitrogen) to allow for nuclear counterstaining. Controls for all immunostainings were performed by omitting the primary antisera. Under these conditions, no staining was observed.

### Morphometric Analysis

Images were acquired by standard epifluorescence using a 0.63X digital camera attached to a Zeiss Axioplan 2 (Carl Zeiss) microscope with a 20X objective. Digitized images of 50–60 neurons were taken of each treatment in replicated cultures. To be photographed for analysis, neurons had to lie separated from adjacent cells so that each process could be clearly attributed to a particular neuron. At DIV3 both the cortical and hypothalamic neurons generally came to lie well scattered throughout the culture dish. Any area where clustered neurons occurred was omitted from the analysis, thus eliminating a biased selection. Morphometric analyses were performed using the MetaMorph Microscopy Automation & Image Analysis software program, Version7.7 (Molecular Devices, Inc., Sunnyvale, CA). Calibration settings were performed and measurement parameters were determined at the onset of the analysis and then maintained throughout the data collection process. Measurements were taken for the total neurite outgrowth, soma size, the number of stem processes per cell originating directly from the cell soma, and the number of neuritic branch points. Data were obtained from 5 independent replicate experiments resulting in a total of 250–300 analyzed cells per treatment. All morphological parameters were calculated and expressed per individual neuron.

### Statistical Analysis

Morphometric comparisons between untreated and treated cells were performed using the non-parametric Kruskal-Wallis one-way analysis of variance followed by the Dunn’s Multiple Comparison test. A level of P < 0.05 was considered to be statistically significant.

## Results

### Time course

**Fig 1:**
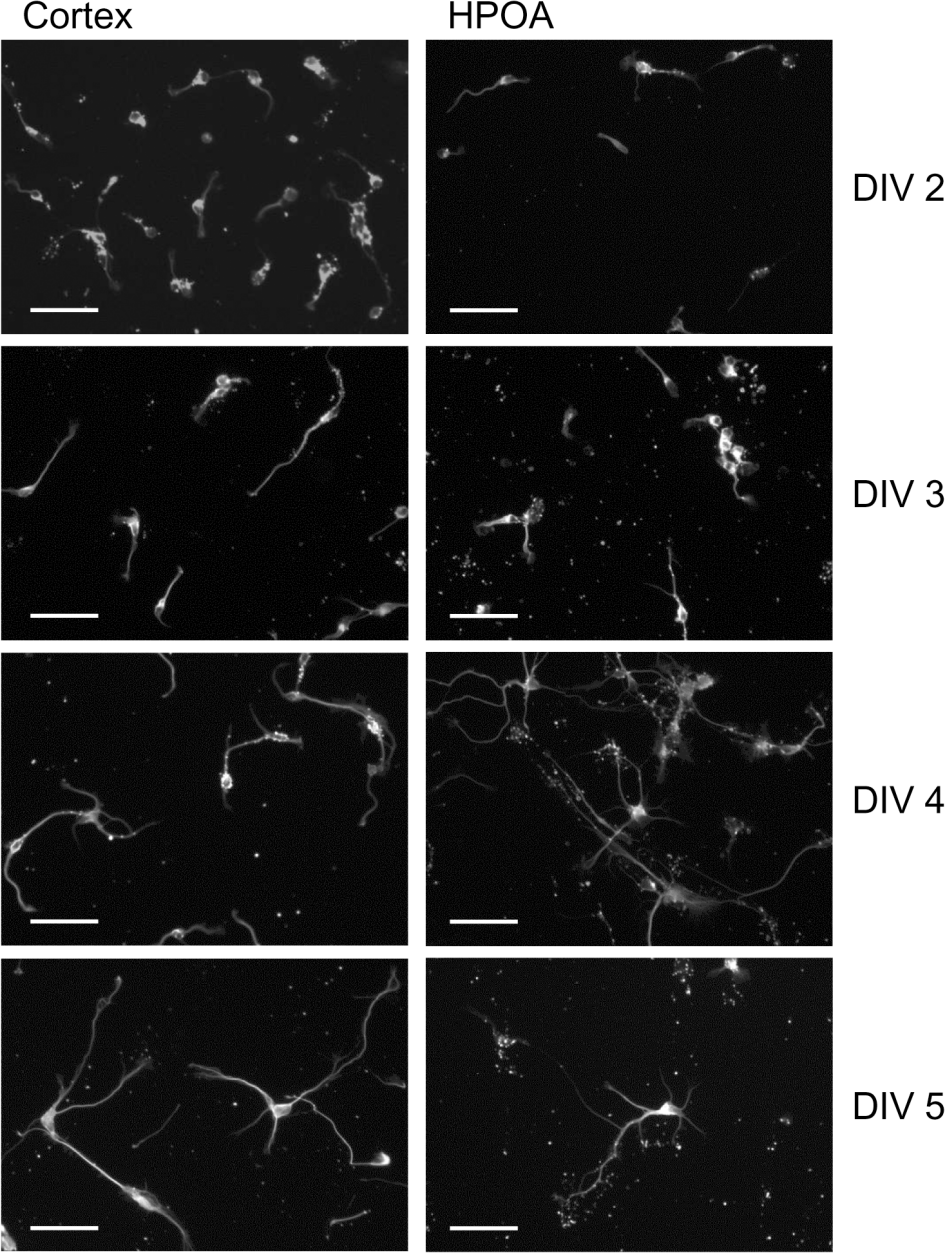
Photomicrographs illustrating the growth of untreated primary neurons derived from GD53 fetal lamb cortex and hypothalamus-preoptic area (HPOA) maintained *in vitro* for 2 days (DIV2) to 5 days (DIV5). Soma and neurites were identified by immunohistochemical staining with the neuron-specific mouse anti-β-tubulin antibody. Scale Bar = 50 μm.

**Fig 2:**
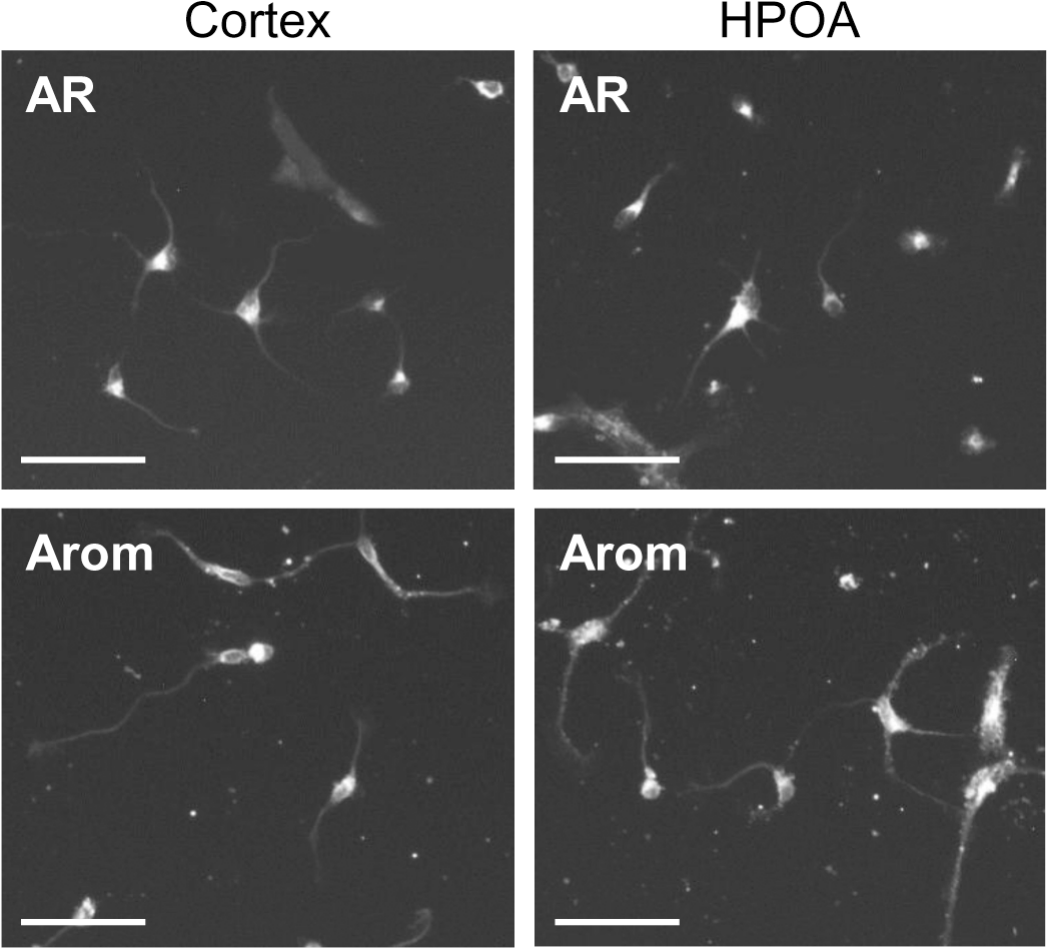
Immunohistochemical staining of untreated cortical and HPOA neurons in cultures grown for 3 days (DIV3). Shown is immunofluorescence labeling for androgen receptor (AR) and aromatase (Arom). Scale Bar = 50 μm.

A time course study was performed on 3 independent replicates with cells derived from the CTX and HPOA of two female and one male lamb fetus. Twenty four hrs after plating cells were attached to the poly-L-lysine substrate and showed neuritic extensions, such as lamellipodia and minor processes (not shown). Neurons were fixed and stained between DIV 2 and 5 (Fig 1). Soma and neurites were identified by cross reactivity with neuron-specific mouse anti-β-tubulin antibody. By DIV2 neurons showed 2 or more primary neurites with little branching. Between DIV 2 and 3 neurites increased in length and began to show branches which further elongated and became more complex with time in culture. Immunocytochemistry for androgen receptor and aromatase revealed positive staining of the cytoplasm and processes of both hypothalamic and cortical β-tubulin-identified neurons at all of the time points examined e.g., DIV 3 shown in Fig 2.

### Effects of testosterone treatment

**Fig 3:**
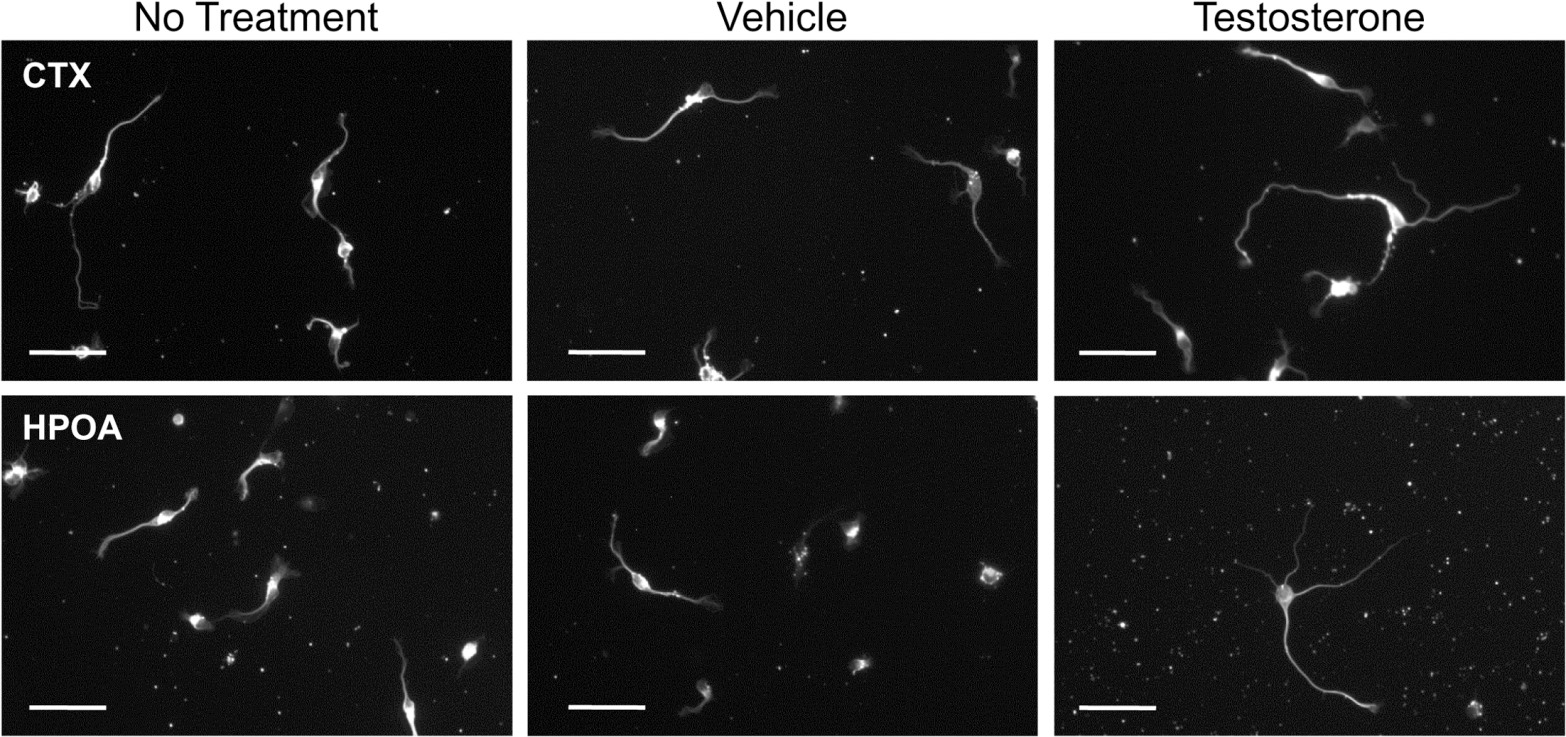
Photomicrographs illustrating the effects of testosterone (10 nM) treatment for 3 days on the morphology of cortical and HPOA neurons immunolabeled for neuron-specific anti-β-tubulin. Morphometric analysis was carried out on randomly selected neurons from each subgroup. In order to control for possible variations between experimental replicates, untreated cells (No treatment) were evaluated together with control (Vehicle) and testosterone-treated cells. Scale Bar = 50 μm.

Cells derived from CTX and HPOA of 5 male lamb fetuses were used in independent replicate experiments to study the effect of testosterone treatment (10 nM) on neuronal differentiation *in vitro* (Fig 3). In order to control for possible variations between experimental replicates, untreated cells were evaluated together with control and testosterone-treated cells. Treatment of cultures with testosterone for 3 days significantly changed morphological parameters related to neuronal differentiation in both CTX and HPOA cells (Table 1). Total neurite outgrowth was significantly increased (P < 0.05) by testosterone treatment in both cortical (21%) and hypothalamic (35%) neurons compared to vehicle controls. Testosterone significantly (P < 0.01) increased soma area in hypothalamic neurons (8%), but not in cortical neurons. The number of branch points was significantly (P < 0.05) higher in testosterone treated cortical neurons, but only showed a trend for hypothalamic neurons (P<0.07). The number of processes was not significantly altered by testosterone in cells derived from either brain region.

**Table 1:** Morphometric Cell Measurements.

Tissue	Cortex				MPOA			
Treatment	No Tx	Veh	T	P*	No Tx	Veh	T	*P*
**Total Outgrowth (um)**	43.3±2.3	51.2±2.8	61.8±3.4	<0.05	30.3±1.9	34.0±2.6	45.9±3.5	0.001
**Soma Size (um2)**	62.5±1.3	63.4±1.3	65.7±1.4	ns	60.3±1.0	63.0±1.2	68.3±1.3	<0.01
**# of Processes**	2.8±.09	2.8±.08	3.0±.09	ns	2.4±.08	2.6±.09	2.8±.1	ns
**# of Branch Pts**	1.0±.11	1.2±.14	1.6±.16	<0.05	.68±.08	.71±.10	1.1±.17	ns(0.067)

Data represent mean ± SEM obtained from 5 independent experiments using cells cultured for 3 days (DIV 3) that were derived from 5 separate GD53 male fetuses. Morphometric comparisons between untreated (No Tx), vehicle- (Veh) and testosterone (T)-treated (10 nM) cells were performed using the non-parametric Kruskal-Wallis one-way analysis of variance followed by the Dunn’s Multiple Comparison test. A level of P < 0.05 was considered to be statistically significant. No significant differences were observed between the No Tx and Veh groups.

*P values indicate measures that are significantly different between the Veh- and T-treated groups. ns = not significantly different (P>0.05).

## Discussion

The present experiments demonstrate that androgens are capable of promoting neurite outgrowth *in vitro* in both cortical and hypothalamic neurons derived from fetal lamb brains. Testosterone enhanced soma area in hypothalamic neurons and promoted branching in cortical neurons. Greater neurite arborization implies the potential for a larger dendritic field and greater afferent input, whereas soma size relates inversely to firing threshold and recruitment potential. The subtle differences in response to testosterone between hypothalamic and cortical neurons suggest that there is regional specificity in the morphogenic potential of androgens on neuronal differentiation. More generally, our results support the conclusion that androgens have the potential to affect the morphological and functional differentiation of the sheep hypothalamus and cortex.

We found that both androgen receptor and aromatase-immunoreactivity was detectable in cortical and hypothalamic neurons. These results indicate that testosterone or its estrogenic metabolite could act directly to influence neuronal maturation, rather than or in addition to indirect effects on their afferent inputs, efferent targets or glial interactions. The presence of androgen receptors and aromatase in the cultures agrees with their expression in the same brain regions of sheep during comparable gestational ages *in vivo* [29–31] and indicates that the culture model has validity for studying steroid hormone action on sheep brain development. The predominant localization of androgen receptor to the cytoplasm may be related to the lack of testosterone in the culture medium during the time course study. Cytoplasmic localization of androgen receptor and aromatase protein has been observed previously in untreated cultured motor neurons [32]. Study of androgen receptor trafficking *in vitro* using a green fluorescent protein-androgen receptor fusion protein demonstrates that the receptor is predominantly cytoplasmic in the absence of hormone and rapidly translocates to the nucleus after testosterone treatment [33].

Our results are generally consistent with previous studies that used primary neuronal cultures derived from rodents [34–39]. These studies found that testosterone promotes neurite outgrowth and other aspects of neuronal maturation in hypothalamic neurons, but less so in cortical neurons. The more robust effect of testosterone on cortical neurons derived from sheep in contrast to rodents might reflect species differences in neuron maturity or steroid sensitivity at the developmental stages examined. The present study also differs methodologically from earlier rodent experiments in the use of a physiological testosterone concentration and shorter length of treatment that began at plating. The observation that effects were apparent by three days in culture suggests that testosterone directly accelerates processes involved in the initiation of dendrite growth and arborization.

The structural effects of testosterone on the developing rodent brain are largely dependent on aromatization of androgens to estrogens. Recent studies have elucidated diverse cellular mechanisms by which estradiol masculinizes the HPOA of rats [40]. However, the effects of androgen and estrogen are intimately entwined and often complimentary. For instance, in mouse neuronal cultures, androgen receptors and aromatase co-exist in the same neurons and testosterone specifically supports differentiation of aromatase-positive hypothalamic neurons, but not cortical or hypothalamic GABAergic neurons [15]. Thus testosterone is not only a substrate for aromatase, but also acts to promote the development of estrogen-producing neurons. Furthermore, the morphogenic effects of testosterone depend on the androgen receptor since they are blocked by the antagonist flutamide [15]. Previous research on *in vitro* PC-12 cell lines transfected with steroid receptor genes suggest that androgen and estrogen act in complimentary and overlapping ways to promote neuronal maturation [41]. Our recent study suggests that the androgen receptor mediates masculinization of the oSDN in sheep [42]. However in addition to androgen receptors, neurons within the developing sheep hypothalamus also express abundant levels of aromatase and estrogen-alpha receptors [17,30,31]. The extent to which estrogen may be involved in brain masculinization in sheep is not currently known. The higher circulating testosterone levels in male lamb fetuses provide the substrates and ligands needed to activate both androgen and estrogen signaling pathways which could in turn lead to sex-specific circuit formation. Moreover, differences in cellular expression of steroid metabolic enzymes and steroid hormone receptor compliment could lead to developmental and regional differences in the effects of gonadal steroids on neuronal morphology and contribute to neuroanatomical and functional sex dimorphisms. Future studies that employ steroid receptor agonists and antagonists are needed to determine the roles and morphogenic potentials that can be attributed to the direct actions of androgens, estrogens or their combination on fetal sheep neurons in culture.

In summary, the present study demonstrates that testosterone stimulates morphological differentiation of cultured fetal lamb neurons derived from the cerebral cortex and hypothalamus by increasing neurite outgrowth and soma size. These data suggest that testosterone acts as a morphogenic signal for developing sheep neurons and by so doing contributes to the maturation of a fully masculine brain. The results extend previous findings obtained in rodents, a species in which sexual differentiation of the brain is completed after birth, to sheep, a long gestation species whose brain is sexually differentiated prior to birth.
